# Macrophage-derived neutrophil chemotactic factor is involved in the neutrophil recruitment inhibitory activity present in the supernatants of LPS-stimulated macrophages

**DOI:** 10.1155/S0962935196000208

**Published:** 1996-04

**Authors:** B. M. Tavares-Murta, F. Q. Cunha, M. Dias-Baruffi, M. C. Roque-Barreira, S. H. Ferreira

**Affiliations:** 1 Department of Pharmacology Faculty of Medicine of Ribeirão Preto USP Ribeirão Preto SP 14049-900 Brazil; 2 Department of Parasitology, Microbiology and Immunology Faculty of Medicine of Ribeirão Preto USP Ribeirão Preto SP 14049-900 Brazil

## Abstract

In a previous study, we demonstrated the presence of a neutrophil recruitment inhibitory factor (NRIF) in the supernatants of LPS-stimulated macrophages. Recently, the purification of a 54 kDa protein, identified as the macrophage-derived neutrophil chemotactic factor (MNCF) was reported. Since NRIF and MNCF are obtained under the same conditions, and, since the intravenous administration of TNF-α and IL-8 inhibits neutrophil migration, we have investigated whether MNCF could be responsible for this inhibitory activity. After affinity chromatography of the macrophage supernatants on a D-galactose column, the inhibitory activity was recovered in both the unbound (D-gal^−^) and bound (D-gal^+^) fractions, with MNCF being found in the D-gal^+^ fraction. Further gel filtration of the latter on Superdex 75 yielded a single peak containing both activities. In a cytotoxicity assay, most of the TNF found in the crude supernatants was recovered in the D-gal^−^ fraction. Furthermore, the incubation of the D-gal^−^ fraction with anti-TNF-α plus anti-IL-8 antisera partially prevents its inhibitory effect on neutrophil migration, but had no effect on the D-gal^+^ activity. Overall, these results suggest that the D-gal^−^ inhibitory effect is partially mediated by TNF-α and IL-8, and that MNCF accounts for the inhibition of neutrophil migration *in vivo* by the D-gal^+^ fraction.


Mediators of Inflammation 5, 116–120 (1996)

### Macrophage-derived neutrophil chemotactic factor is involved in the neutrophil recruitment inhibitory activity present in the supernatants of LPS-stimulated macrophages

B. M. Tavares-Murta,[1] F. Q. Cunha,[1] M. Dias-Baruffi,[2] M. C. Roque-Barreira[2] and S. H. Ferreira[1,CA]

[1]Department of Pharmacology; Fax: (+55) 166 232792; and [2]Department of Parasitology, Microbiology and Immunology, Faculty of Medicine of Ribeirão Preto, USP, 14049-900 Ribeirão Preto, SP, Brazil

[CA]Corresponding Author

**IN a previous study, we demonstrated the presence of a neutrophil recruitment inhibitory factor (NRIF) in the supernatants of LPS-stimulated macrophages. Recently, the purification of a 54 kDa protein, identified as the macrophage-derived neutrophil chemotactic factor (MNCF) was reported. Since NRIF and MNCF are obtained under the same conditions, and, since the intravenous administration of TNF-α and IL-8 inhibits neutrophil migration, we have investigated whether MNCF could be responsible for this inhibitory activity. After affinity chromatography of the macrophage supernatants on a D-galactose column, the inhibitory activity was recovered in both the unbound (D-gal⁻) and bound (D-gal⁺) fractions, with MNCF being found in the D-gal⁺ fraction. Further gel filtration of the latter on Superdex 75 yielded a single peak containing both activities. In a cytotoxicity assay, most of the TNF found in the crude supernatants was recovered in the D-gal⁻ fraction. Furthermore, the incubation of the D-gal⁻ fraction with anti-TNF-α plus anti-IL-8 antisera partially prevents its inhibitory effect on neutrophil migration, but had no effect on the D-gal⁺ activity. Overall, these results suggest that the D-gal⁻ inhibitory effect is partially mediated by TNF-α and IL-8, and that MNCF accounts for the inhibition of neutrophil migration *in vivo* by the D-gal⁺ fraction.**

**Key words**: Cytokine, inflammation, inhibition, leukocyte, migration, neutrophil

### Introduction

Several studies have established that Gram-negative bacteraemia or circulating endotoxin decreases the ability of neutrophils to migrate into the inflammatory sites, a phenomenon which may play an important role in the evolution of sepsis.[1–3] It has been demonstrated that the impairment of neutrophil migration is mediated by inhibitory factors including tumour necrosis factor-α (TNF-α)[4] and interleukin 8 (IL-8).[5]

Previous work from our laboratory demonstrated that the intravenous administration of LPS-stimulated macrophage supernatants also inhibits neutrophil migration to the inflammatory site following exposure to various stimuli.[6] When the supernatants were submitted to gel filtration on Sephacryl S-300, the *in vivo* neutrophil chemotactic inhibitory activity was detected mainly in a fraction which eluted in a volume corresponding to high molecular weight proteins (240–550 kDa). This fraction also inhibited the cell-dependent oedema induced by carrageenin or by ovalbumin in previously immunized rats. The cell-independent oedema induced by dextran was not affected by the S-300 active fraction.[7] This fraction was also able to inhibit the increase in neutrophil and eosinophil numbers in the bronchoalveolar lavage fluid of sensitized guinea-pigs as well as the ovalbumin-induced bronchoconstriction in these animals.[8]

Recently, the purification of a 54 kDa acidic protein, identified as the macrophage-derived neutrophil chemotactic factor (MNCF) was reported.[9] The *in vivo* chemotactic activity of MNCF in animals pretreated with dexamethasone is an uncommon characteristic which discriminates MNCF from known chemotactic cytokines, such as TNF-α and IL-8. MNCF induces neutrophil migration through a carbohydrate-recognizing property.[10]

Since NRIF and MNCF are obtained under the same *in vitro* conditions, i.e. in the supernatants of LPS-stimulated macrophages, MNCF activity is also detected in the S-300 active fraction,[7] we have investigated whether MNCF account, at least in part, for the NRIF activity. For this, we have

© 1996 Rapid Science Publishers purified macrophage supernatants using a simple two-step process involving adsorption to a D-galactose column followed by gel filtration on Superdex 75. We have demonstrated that TNF-α and IL-8 mediate the activity of the D-gal$^-$ fraction and that MNCF is responsible for the *in vivo* neutrophil recruitment inhibitory activity of the D-gal$^+$ fraction.

### Materials and Methods

*Animals:* Male Wistar rats weighing 180 to 200 g and housed in a temperature-controlled room received water and food *ad libitum*. The rats were used as the source of peritoneal macrophages as well as for the *in vivo* assays of neutrophil migration inhibition.

*Production of macrophage supernatants:* The method for obtaining crude macrophage supernatants containing the inhibitory activity has been described in detail.[4] Briefly, rat macrophages were harvested from peritoneal cavities elicited 4 days earlier with 10 ml of 3% thioglycollate (w/v) and incubated in tissue culture dishes for 1 h at 37°C, in an atmosphere of air containing 5% $CO_2$. The adherent monolayers were washed three times with sodium phosphate-buffered saline (PBS, pH 7.4) and incubated with LPS (5 μg/ml of RPMI) for 30 min at 37°C. The cells were again washed three times with PBS followed by a final incubation with LPS-free medium, for 90 min at 37°C. The cell-free incubation medium was centrifuged (2 000 × **g** for 10 min at 25°C) and subsequently ultrafiltered through a YM-10 membrane (Amicon Corp., Lexington, MA, USA) against sterile, deionized water at 4°C. The supernatants were then concentrated to 5 ml and filtered through a 0.22 μm membrane (Millipore, Bedford, MA, USA). This preparation was designated as the crude supernatant, and was either used for *in vivo* or *in vitro* experiments, or was chromatographed on an agarose/D-galactose column.

*Chromatographic procedures for the fractionation of crude supernatant:* Partial purification of the crude supernatant was performed as described by Dias-Baruffi *et al.*[9] Briefly, crude supernatant obtained from 3.6 × $10^8$ adherent cells was chromatographed on an agarose/D-galactose column (Pierce Chemical Co., Rockford, IL, USA) at 4°C. The material not retained by the column (D-gal$^-$) was eluted with sterile water, and the adsorbed substance (D-gal$^+$) was eluted with 0.4 M D-galactose. The two fractions were ultrafiltered through YM-10 membranes and used in the experiments described below. In some cases, the D-gal$^+$ fraction was further chromatographed on a Superdex 75 column. For this, the fraction was concentrated to 0.2 ml and applied to a Superdex 75 HR 10/30 column (Pharmacia LKB Biotechnology, Uppsala, Sweden) previously equilibrated with PBS and calibrated with known molecular weight markers. Fractions of 0.5 ml were collected and tested *in vivo* or *in vitro*.

In vivo *neutrophil migration assay:*

*Inhibition assay.* Crude supernatant, D-gal$^+$, D-gal$^-$ or Superdex 75 chromatographic fractions were injected intravenously (i.v.) into the penial venous sinus of rats. Each animal received material equivalent to that released by a 6 × $10^6$ macrophages in 0.2 ml. Thirty min later, the animals received an intraperitoneal (i.p.) injection of carrageenin (300 μg/ml) (Marine Colloids, Inc., USA) while the controls (C) received PBS. After 4 h, the animals were sacrificed and their peritoneal cavities washed with 10 ml of PBS containing heparin (5 IU/ml). Total and differential cell counts were performed as described elsewhere,[11] and the results were expressed as the percentage (%) of inhibition.

*Effect of anti-TNF-α and anti-IL-8 antisera on the inhibition of neutrophil migration caused by the D-gal$^-$ and D-gal$^+$ fraction.* The D-gal$^-$ or D-gal$^+$ fraction (obtained from 6 × $10^6$ cells) was incubated with sheep antisera (20 μl/200 μl of sample) against murine recombinant TNF-α (mrTNF-α) and against human recombinant IL-8 (hrIL-8) in a 5% $CO_2$ atmosphere at 37°C, for 30 min. As a control, the fractions were also incubated with normal serum. Subsequently, each fraction was administered i.v., 30 min before the i.p. injection of carrageenin (300 μg). After 4 h, total and differential cell counts were performed[11] and the results were expressed as the number of neutrophils × $10^6$/ml of lavage fluid.

*TNF activity assay.* The TNF content in the crude supernatant and in the chromatographic fractions was measured using a highly TNF-sensitive cell line, WEHI 164 clone 13, as described elsewhere.[12] Briefly, WEHI cells were seeded in microplates (Costar 3596, Cambridge, MA, USA) at concentrations of 4 × $10^4$ cells/well in 50 μl of RPMI containing 10% foetal calf serum. Thirty μl of the samples were added in quadruplicate to the cells. In some experiments, we used a rabbit anti-mrTNF-α antibody to study the involvement of TNF-like cytokines in our samples. In these cases, 20 μl of the antibody were added to the cells immediately before the addition of the

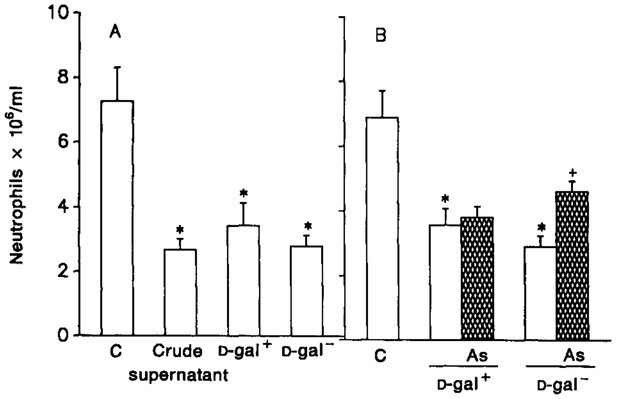

FIG. 1. Inhibitory action of crude supernatant and D-galactose fractions on neutrophil migration and the effect of anti-TNF-α and anti-IL8 antisera. The columns indicate the neutrophil migration induced 4 h after the i.p. administration of carrageenin (300 μg) in rats pretreated (30 min) with i.v. injections of PBS (C, control), LPS-stimulated macrophage supernatants (crude supernatant), and D-gal$^+$ or D-gal$^-$ fractions incubated with (panel B) or without (panel A) the antisera (As). In panel B, the D-galactose fractions were co-incubated with sheep antisera (20 μl/200 μl of sample) against mrTNF-α and hrIL-8 for 30 min. In both panels, the crude supernatant or D-galactose fractions were obtained from 6 × $10^6$ macrophages. Panel A: *$p < 0.01$ compared to control (ANOVA + Bonferroni). Panel B: *$p < 0.01$ compared to control, + $p < 0.05$ compared to D-gal$^-$ alone (ANOVA + Student's *t*-test).

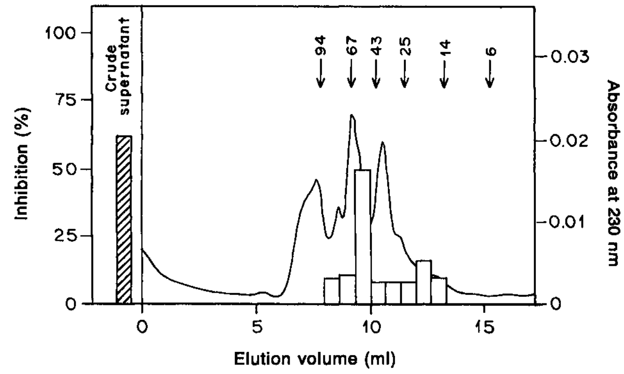

FIG. 2. Effect of Superdex 75 fractions on the inhibition of neutrophil migration. The D-gal$^+$ fraction obtained from 3.6 × $10^8$ macrophages was applied to a Superdex 75 column. Fractions of 0.5 ml eluted in the volume range corresponding to proteins of 14–90 kDa were collected and assayed *in vivo*. The columns represent the neutrophil migration induced 4 h after carrageenin (300 μg, i.p.) in rats pretreated (30 min) with i.v. injections of the corresponding fraction (material obtained from 1.2 × $10^7$ macrophages/0.2 ml). Controls received PBS or crude supernatant. The chromatographic profile was determined at 230 nm. *$p < 0.01$ compared to PBS (ANOVA + Student's *t*-test).

samples. The plates were incubated for 20 h at 37°C in a 5% $CO_2$ incubator, after which 10 μl of a 3-4,5-dimethylthiazol-2-yl-3,5-diphenylformazan solution (5 mg/ml of PBS) (MTT, Sigma) were added to each well and the plates incubated for an additional 4 h. Subsequently, 100 μl of isopropanol containing 0.04 N HCl were added to each well. Fifteen min later, the degree of cell lysis was quantitated spectrophotometrically (570 nm) by using an enzyme-linked immunoassay analyser (Multiskan MCC/340 MKII, Flow Laboratories). Because of the unavailability of rat TNF-α, standard curves were prepared with mrTNF-α (Genentech Inc.) and the TNF content in the assayed samples was calculated by comparison with the standard amounts. The results were expressed as the percentage (%) of cell death and correspond to the mean of data obtained in two different experiments.

*Statistical analysis:* The results are expressed as the means ± standard error of the means (S.E.M.) of five animals in each case. The results are representative of two or three different experiments. Statistical analysis was performed by analysis of variance followed by the Bonferroni or Student's *t*-test. Statistical differences were considered significant for $p < 0.05$.

### Results

*Inhibitory action of crude supernatant and D-galactose fractions on neutrophil migration and the effect of anti-TNF-α and anti-IL-8 antisera:* Figure 1(A) confirms our previous results by demonstrating that LPS-stimulated macrophage supernatants, when injected intravenously, inhibit neutrophil migration. After chromatography of the crude supernatants on an agarose/D-galactose column, the D-gal$^+$ and D-gal$^-$ fractions inhibited carrageenin-induced neutrophil migration into rat peritoneal cavities.

When the D-gal$^-$ fraction was co-incubated with anti-TNF-α plus anti-IL-8 antisera, its inhibitory activity was partially prevented. In contrast, the same treatment did not alter the inhibitory effect of the D-gal$^+$ fraction on neutrophil migration (Fig. 1(B)).

*Effect of Superdex 75 fractions on the inhibition of neutrophil migration:* The D-gal$^+$ preparation was chromatographed on a Superdex 75 column and the ability of some of the fractions to inhibit neutrophil migration *in vivo* was tested. Figure 2 shows that the inhibitory activity present in the crude supernatant was detected in the fraction eluted in a volume of 10 ml, corresponding to an apparent molecular weight of 54 kDa, as shown by the chromatographic profile. In previous work, this fraction was also demonstrated to contain neutrophil chemotactic activity. Electrophoretic analysis of the D-gal$^-$ fraction showed

**Table 1.** Cytotoxic activity of the crude supernatant and chromatographic fractions on WEHI cells

| Treatment | Samples | Cell death (%) |
|---|---|---|
| | Crude supernatant | 61.5 |
| RPMI | D-gal⁻ | 50.7 |
| | D-gal⁺ | 7.6 |
| | Fraction 20 | 3.4 |
| | Crude supernatant | 15.5 |
| Antiserum | D-gal⁻ | 7.2 |
| anti-mrTNF-α | D-gal⁺ | 1.6 |
| | Fraction 20 | 0 |

The results are expressed as the percentage of cell death after the treatment of WEHI cells 164 clone 13 with LPS-stimulated macrophage supernatants (crude supernatant), D-gal⁻ or D-gal⁺ fractions, or the active fraction of the Superdex 75 chromatography (corresponding to fraction 20, at an elution volume of 10 ml), in the presence of anti-mrTNF-α antibody or RPMI medium. Fraction 20 obtained after chromatography of the D-gal⁺ fraction on Superdex 75 was not cytotoxic to WEHI cells.

multiple bands, in contrast to the D-gal$^+$, in which were found four protein bands of 39, 45, 54 and 68 kDa. Further electrophoresis of the active fraction (elution volume of 10 ml) showed only a single protein band corresponding to 54 kDa.[9]

*TNF cytotoxic activity in the crude supernatant and chromatographic fractions:* When tested for TNF cytotoxic activity using WEHI cells, the crude supernatant contained a high amount of this cytokine (killing 61.5% of the cells) which was recovered mainly in the D-gal$^-$ fraction. In the D-gal$^+$ fraction, only small amounts of TNF were found (7.6% of cells killed), and this decreased to almost zero after chromatography on Superdex 75. Antibodies raised against mrTNF-α abolished the cytotoxicity of mrTNF-α (data not shown) and strongly reduced the activities of the crude supernatant and the D-gal$^-$ fraction, thus confirming that the cytotoxic activity observed was due to TNF (Table 1).

### Discussion

In the present paper, we have confirmed previous results showing that the intravenous administration of LPS-stimulated macrophage supernatants inhibits the neutrophil migration induced by carrageenin, LPS or *Pseudomonas aeruginosa*.[6] An affinity chromatographic procedure using a D-galactose affinity column showed that the inhibitory activity was recovered in both the bound and unbound fractions.

The inhibitory effect presented by the D-gal$^-$ fraction was due, at least in part, to the presence of TNF-α, since a significant amount of this cytokine was found in this fraction. In support of this, incubation of the D-gal$^-$ fraction with anti-TNF-α plus anti-IL-8 antisera partially prevented the inhibitory action. In this context, TNF-α has been reported to inhibit neutrophil migration in different animal models.[4,13] The anti-IL-8 antiserum was employed because we have previously detected IL-8 in samples of crude LPS-stimulated macrophage supernatants (data not shown), and this cytokine is also known to inhibit neutrophil migration.[5,13,14] In contrast, the D-gal$^+$ effect was not due to contamination with TNF or even to the presence of IL-8, since no TNF was detected in the Superdex 75 fraction, nor did the anti-TNF plus anti-IL-8 antisera have any effect on the ability of the fraction to inhibit neutrophil migration.

Following further gel infiltration of the D-gal$^+$ fraction on Superdex 75, the inhibitory activity was recovered in a single fraction equivalent to a molecular weight of 54 kDa. Recently, we described the purification of a 54 kDa acidic protein, identified as MNCF.[9] This protein causes *in vitro* chemotaxis as well as *in vivo* neutrophil migration even in animals treated with dexamethasone.[10] Interestingly, in the present study, the neutrophil inhibitory activity was located in the same chromatographic fraction as that in which MNCF was previously detected, suggesting that the same protein accounts for both chemotactic (MNCF) and inhibitory (NRIF) activities. These results are consistent with literature data which have already demonstrated that the pro-inflammatory chemotactic cytokines TNF-α and IL-8, when administered i.v., are able to inhibit the neutrophil recruitment to the inflammatory site.[4,5,13,14]

Our results suggest that the inhibitory effect of the D-gal$^-$ fraction is mediated by TNF-α and IL-8. Moreover, in relation to the D-gal$^+$ fraction, the chemotactic and inhibitory activities on neutrophil migration are due to the same protein, already identified as a 54 kDa acidic protein. The mechanisms by which TNF-α, IL-8 and MNCF inhibit neutrophil migration are currently under investigation.

### References

1. Smith MJH, Ford-Hutchinson AW, Walker JR. Anti-inflammatory activity of bacterial endotoxin. *J Pharm Pharmacol* 1977; **29**: 702–704.
2. Van Dijk WC, Verbrugh HA, Van der Tol ME, Peters R, Peterson PK, Quie PG, Verhoef J. Interactions of phagocytic and bacterial cells in patients with bacteremia caused by Gram-negative rods. *J Inf Dis* 1980; **141**: 441–448.
3. Rocha NP, Ferreira SH. Restoration by levamisole of endotoxin inhibited neutrophil migration, oedema and increased vascular permeability induced by carrageenin. *Eur J Pharmacol* 1986; **122**: 87–92.
4. Otsuka Y, Nagano K, Nagano K, *et al.* Inhibition of neutrophil migration by tumor necrosis factor. *Ex vivo* and *in vivo* studies in comparison with *in vitro* effect. *J Immunol* 1990; **145**: 2639–2643.
5. Hechtman DH, Cybulsky MI, Fuchs HJ, Baker JB, Gimbrone MA. Intravascular IL-8. Inhibitor of polymorphonuclear leukocyte accumulation at sites of acute inflammation. *J Immunol* 1991; **147**: 883–892.
6. Cunha FQ, Souza GEP, Souza CAM, Cerqueira BCS, Ferreira SH. *In-vivo* blockage of neutrophil migration by LPS is mimicked by a factor released from LPS-stimulated macrophages. *Br J Exp Pathol* 1989; **70**: 1–8.

7. Tamashiro WMSC, Tavares-Murta BM, Cunha FQ, Nogueira RMD, Roque-Barreira MC, Ferreira SH. Neutrophil recruitment inhibitory factor: a possible candidate for a novel cytokine. *Med Inflamm* 1992; **1**: 49–54.
8. Tavares-Murta BM, Lefort J, Cunha FQ, Ferreira SH, Vargaftig BB. Interference of a neutrophil recruitment inhibitory factor upon the accumulation of inflammatory cells and airway hyperreactivity in sensitized guinea-pigs after intranasal antigen challenge. *Br J Pharmacol* 1993; **108**: 538–543.
9. Dias-Baruffi M, Roque-Barreira MC, Cunha FQ, Ferreiera SH. Isolation and partial characterization of macrophage-derived neutrophil chemotactic factor. *Med Inflamm* 1995; **4**: 257–262.
10. Dias-Baruffi M, Roque-Barreira MC, Cunha FQ, Ferreiera SH. Biological characterization of purified macrophage-derived neutrophil chemotactic factor. *Med Inflamm* 1995; **4**: 263–269.
11. Souza GEP, Ferreira SH. Blockade by antimacrophage serum of the migration of PMN neutrophils into the inflamed peritoneal cavity. *Agents Actions* 1985; **17**: 987–103.
12. Spevik T, Nissen-Meyer J. A highly sensitive cell line, WEHI 164 clone 13, for measuring cytotoxic factor/tumor necrosis factor from human monocytes. *J Immunol Methods* 1986; **95**: 99–105.
13. Cunha FQ, Tamashiro WMSC. Tumour necrosis factor-alpha and interleukin-8 inhibit neutrophil migration *in vitro* and *in vivo*. *Med Inflamm* 1992; **1**: 397–401.
14. Ley K, Baker JB, Cybulsky MI, Gimbrone Jr MA, Luscinskas FW. Intravenous interleukin-8 inhibits granulocyte emigration from rabbit mesenteric venules without altering L-selection expression or leukocyte rolling. *J Immunol* 1993; **151**: 6347–6357.

ACKNOWLEDGEMENTS. This work was supported by FAPESP and CNPq. B.M.T-M is on leave from the Departamento de Ciências Biológicas, Faculdade de Medicina do Triângulo Mineiro, Uberaba, MG, and is supported by a grant from CNPq. The authors thank Neomesia I. S. Freiri, Ana Kátia dos Santos, Fabíola Mestriner and Sandra M. O. Thomaz for the expert technical assistance.

**Received 31 January 1996; accepted 15 February 1996**
